# Energy Efficient Link Aware Routing with Power Control in Wireless Ad Hoc Networks

**DOI:** 10.1155/2015/576754

**Published:** 2015-06-08

**Authors:** Jeevaa Katiravan, D. Sylvia, D. Srinivasa Rao

**Affiliations:** ^1^Department of Information Technology, Velammal Engineering College, Chennai 600 066, India; ^2^Department of ECE, Jawaharlal Nehru Technological University, Hyderabad 500 085, India; ^3^Department of ECE, Velammal Engineering College, Chennai, India

## Abstract

In wireless ad hoc networks, the traditional routing protocols make the route selection based on minimum distance between the nodes and the minimum number of hop counts. Most of the routing decisions do not consider the condition of the network such as link quality and residual energy of the nodes. Also, when a link failure occurs, a route discovery mechanism is initiated which incurs high routing overhead. If the broadcast nature and the spatial diversity of the wireless communication are utilized efficiently it becomes possible to achieve improvement in the performance of the wireless networks. In contrast to the traditional routing scheme which makes use of a predetermined route for packet transmission, such an opportunistic routing scheme defines a predefined forwarding candidate list formed by using single network metrics. In this paper, a protocol is proposed which uses multiple metrics such as residual energy and link quality for route selection and also includes a monitoring mechanism which initiates a route discovery for a poor link, thereby reducing the overhead involved and improving the throughput of the network while maintaining network connectivity. Power control is also implemented not only to save energy but also to improve the network performance. Using simulations, we show the performance improvement attained in the network in terms of packet delivery ratio, routing overhead, and residual energy of the network.

## 1. Introduction

Wireless ad hoc networks have a wide variety of applications which include disaster management, emergency rescue operations, monitoring, and surveillance. They command vast deployment due to the lack of infrastructure requirement and easy deployability. But these ad hoc networks suffer in terms of two major resources, energy and bandwidth. Most of the devices are battery powered and the optimal utilization of the power becomes a critical issue. It becomes important to implement an energy aware scheme that would achieve a better network performance.

Routing in wireless ad hoc networks is a demanding issue especially while considering the unreliable wireless links and the rapidly diminishing node energy. The traditional routing protocols [1, 2] discover a single fixed path between the source and destination that mainly considers the distance metric and the hop count. But the wireless ad hoc network is subject to frequent link breaks, due to the mobility of the nodes and the depleted energy level. It becomes important to include the nature of the wireless channel and the energy levels while making routing decisions.

Opportunistic routing [3] makes use of the broadcast nature of the medium in which the packets are broadcasted instead of the unicast packets. All the nodes that are within the transmission radius of the sending node coordinate to select the best possible relay. Biswas and Morris [4] proposed ExOR which adopts batch transmission of packets, uses the Expected Transmission Count as the metric, and works better than traditional routing schemes but suffers from duplicate packet transmission. The authors Hsu et al. [5] proposed a token coordination mechanism, in which the packet can be transmitted only by the token holder and prevented duplicate packet transmission but suffered from extra control overhead. Ahlswede et al. [6] avoided duplicate transmissions by including network coding with routing. The authors in [7] have proposed a routing protocol that considers the quality of the communication links while selecting the route.

Most wireless nodes are aware of location information and geographical routing protocols such as Greedy Perimeter Stateless Routing [8], Geographic Random Forwarding [9], and Beacon Less Routing [10] make use of their location information to make routing decisions.

As many forwarding nodes are involved, the probability of correct transmission through at least one forwarding node increases when compared to the conventional routing schemes. It has been shown in literature [11] that the throughput capacity of the network can be greatly increased since the forwarding reliability increases.

Tang et al. [12] analyze the relation between the data rate and progress in a fading scenario and have considered a new metric called bit transfer speed which is the ratio of the progress towards destination and the time required for transfer of data.

There has been extensive work done in the area of power control schemes [13–15]. Jantti and Kim [16] have achieved improvement in the lifetime of the network with the implementation of joint routing and variable rate transmissions.

Most of these schemes have shown that optimization of transmit power level would increase the lifetime of the network and increase the spatial reuse. In this paper, we first propose a novel energy and link quality aware routing scheme which selects optimal forwarding candidates. The route discovery also includes calculation of optimal transmit power by classifying the nodes into clusters based on the transmission radius. Constant link monitoring also ensures route discovery before the failure of the node. To address these issues, a novel approach is proposed in this paper which considers energy and link quality during the routing decisions. This paper proposes a new Energy Efficient Link Quality Aware Routing Protocol with Variable Power Control (ELRPP) for wireless ad hoc networks.

The contributions of this paper are as follows.

- The problem of energy efficient routing is addressed, by taking the SNR, link quality, and residual energy into consideration.
- A variable power level is used for packet transmission based on the distance between nodes, which results in minimization of the power consumption, leading to more residual energy, thereby increasing the lifetime of the network.
- A constant link monitoring with error notification addresses the issue of high overhead incurred due to the usage of periodic control packets.

## 2. Materials and Methods

The lifetime of a wireless network can be extended by proper selection of relay nodes that act as forwarding candidates. The selection of a nearby short hop neighbour would be a better choice than a long distance neighbour, since it would consume more power. In this protocol, a relay node is selected from a set of potential forwarding candidates based on multiple metrics of link quality and residual energy which is an extension of the traditional AODV protocol that works on a single distance metric. A variable adaptive transmit power level is used for packet transmission based on the range of distance of the destination node.

**Figure 1:**
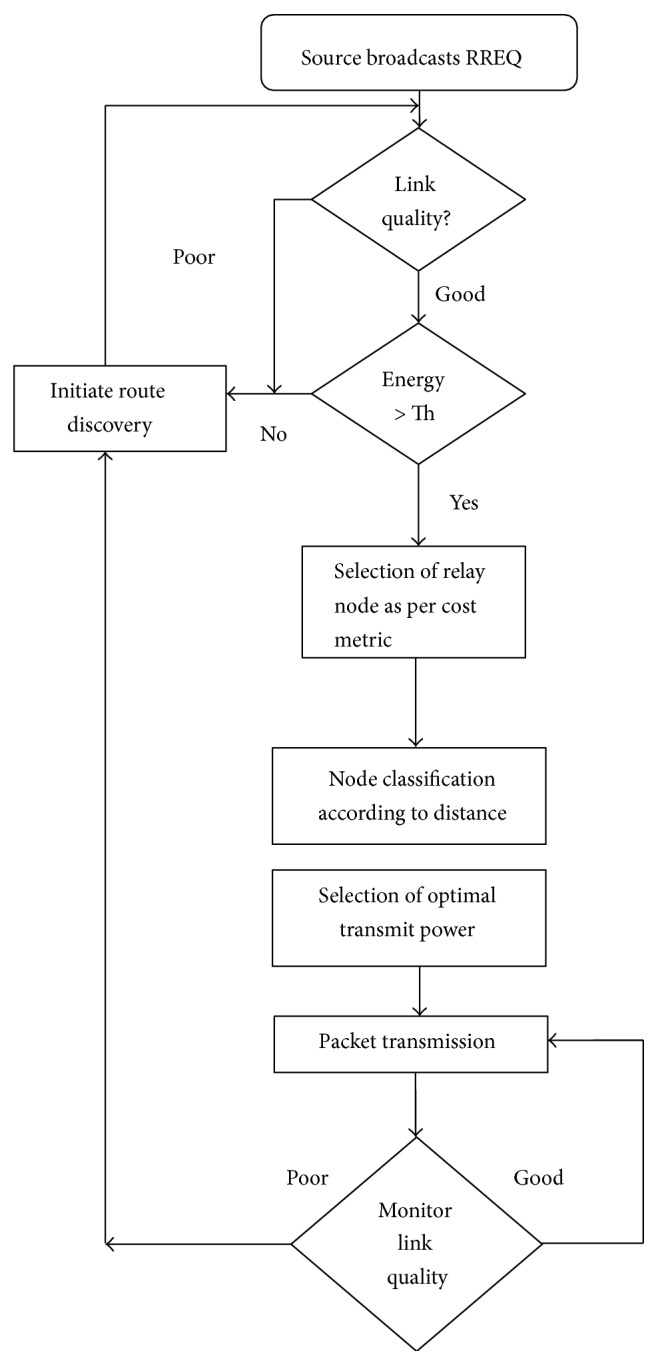
Flow diagram of proposed methodology.

The flow diagram of the proposed methodology is shown in Figure 1. The algorithm is divided into computation of selection metric, selection mechanism, monitoring of link failure, and implementation of power control.

### 2.1. Computation of Selection Metric

A source node broadcasts a packet which contains the topological information and the final destination. All the nodes that receive the packet are potential forwarding candidates. We propose a Cost Function (CF) based on multiple metrics, Link Quality (LQ), and Available Energy (AE). The Cost Function is calculated as

(1) $$\begin{array}{c} \text{CF}=\alpha \cdot \text{LQ}+\beta \cdot \text{AE}, \end{array}$$

where *α* and *β* are the weights assigned to each metric with *α* + *β* = 1. Depending on requirements different weights may be assigned to the metrics.

The link quality should be a measure of the actual channel conditions and the effects of mobility and fading are to be considered. Each node computes the neighbour nodes signal to noise ratio adaptively similar to [17]

(2) $$\begin{array}{c} {\text{snr}}_{\text{inst}}=\gamma \cdot {\text{snr}}_{\text{inst}}+\left(\;1-\alpha \;\right)\cdot {\text{snr}}_{\text{ave}}, \end{array}$$

where snr_inst_ is the current SNR, snr_ave_ is the cumulative SNR averaged over a period, and *γ* is a parameter between 0 and 1. For a high value of *γ*, the effect of the instantaneous SNR is more in the computation of the SNR value.

When the SNR falls below a critical threshold, it returns a metric of 1 and when the SNR is above the threshold required for successful packet transmission it returns a metric of 0, as a measure of link quality:

(3) LQ=1if  SNRinst≥SNRth,LQ=0if  SNRinst<SNRth.

Energy resources in wireless ad hoc networks are scarce and therefore an efficient routing scheme should be energy efficient. If a node has high energy, it returns a small value to the cost function:

(4) AE=Einit−EavlEinitif  Eavl>Emin,AE=1if  Eavl<Emin,

where *E*_init_ is the initial energy, *E*_avl_ is the energy available in the nodes, and *E*_min_ is the minimum threshold energy required for packet transmission.

Thus, a node can be selected as a forwarder when its link quality is good and there is enough available energy in the node.

### 2.2. Selection Mechanism

During the route discovery, the sending node broadcasts a route request message which includes an additional field, Cost Function in the packet header. The node that returns the least Cost Function is chosen as the forwarding relay.

### 2.3. Monitoring of Link Failure

In the existing protocols, such as AODV, periodic hello messages are sent to monitor link failure. When hello packets are not returned within a specific time interval, the nodes remove the entry in the routing table and also send a Route Error packet to all the nodes that have an entry for this link. A route discovery process is initiated on receiving this error message and results in high routing overhead. The packet transmission is disturbed till a new route is discovered. If an error message could be sent before the link completely fails, it would be possible to find a new route before the occurrence of the failure. In our scheme instead of using hello packets, since the SNR value is being continuously computed by the nodes, whenever the link quality falls below a threshold, the node sends an error notification message and thereby maintains the network connectivity.

### 2.4. Implementation of Power Control

The IEEE802.11 MAC is energy inefficient since it uses the same transmission power for packet transmission irrespective of the distance between the sending and receiving nodes. This also reduces the spatial reuse and fairness in the network. Existing protocols compute the optimal power required for the packet transmission between the communicating nodes. This might incur significant overhead. In our method, we first organize the nodes based on their distance from the transmitting node. Three different power levels are used for packet transmission depending on the position of the receiving node.

### 2.5. Pseudocode for the Proposed Algorithm

**Step 1 .**

 Source node creates modified RREQ packet with cost function field.
 Source node broadcasts the RREQ packet to the neighboring nodes.

**Step 2 .**

 Relay nodes compute cost function:
  (5) $$\begin{array}{c} {\text{snr}}_{\text{inst}}=\gamma \cdot {\text{snr}}_{\text{inst}}+\left(\;1-\alpha \;\right)\cdot {\text{snr}}_{\text{ave}}. \end{array}$$
 If SNR_inst_ ≥ SNR_th_,
  (6) $$\begin{array}{c} \text{LQ}=1; \end{array}$$
 else if
 SNR_inst_ < SNR_th_
  (7) $$\begin{array}{c} \text{LQ}=0. \end{array}$$
 Measure available energy
  (8) AE=Einit−EavlEinitif  Eavl>Emin,AE=1if  Eavl<Emin,CF=α·LQ+β·AE.
 Relay nodes enter the CF value in the RREP packet.

**Step 3 .**

 Source node receives route reply packets.
 Choose node with min cost function as relay node.

**Step 4 .**

 Source node chooses power level for transmitting nodes.
 If node distance ≤ transmission radius 1.
 Set transmit power = power level 1.
 Else if node distance ≤ transmission radius 2.
 Set transmit power = power level 2.
 If node distance ≠ transmission radius 1 or 2.
 Set transmit power = max power.

**Step 5 .**

 Monitor link for failure.
 When Sprints < SNRThrsh.
 Relay node sends error notification message to source.
 Source initiates route discovery process.

## 3. Results and Discussion

The proposed scheme has been tested through simulation against the existing protocol AODV [1]. The NS-2 simulator has been used and we deployed 40 nodes randomly in a flat topology of 1000 × 1000 m. The constant CBR traffic of 512 bytes is generated at a constant data rate of 11 Mbps and the nodes are assumed to transmit at a max power level of 15 dBm, with variable power level of 10 dBm and 12 dBm. The nodes are assumed to be mobile using random way point model, and the effect of mobility is considered by a node speed of 20 m/s and the pause time varied from 0 to 50 m/s. The frequency of node movement can be varied by varying the pause time. The path loss exponent is set as 4. The MAC protocol is IEEE802.11b and an omnidirectional antenna with two-ray ground propagation model is chosen. The simulation is run for 100 s and the radio transmission range is 250 m.

**Figure 2:**
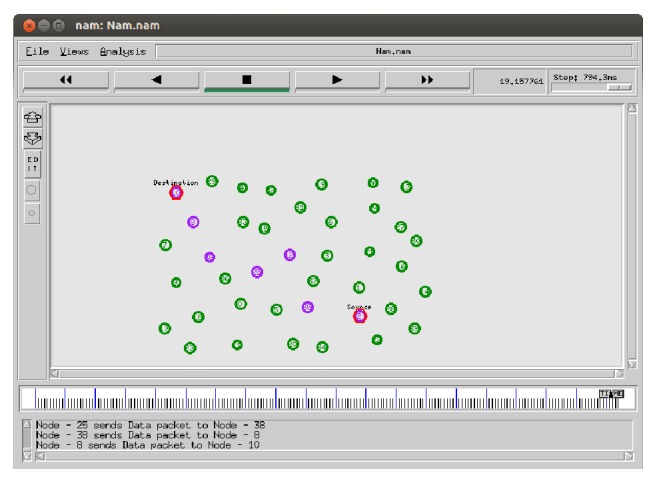
Simulated network.

The simulated network is shown in Figure 2.

Throughput and end-to-end delay are the most important metrics for evaluating best-effort traffic and the routing overhead evaluates the efficiency of the routing protocol [18].

### 3.1. Packet Delivery Ratio

It is defined as the number of data packets delivered to the destination to the number of packets generated by the CBR traffic source.

**Figure 3:**
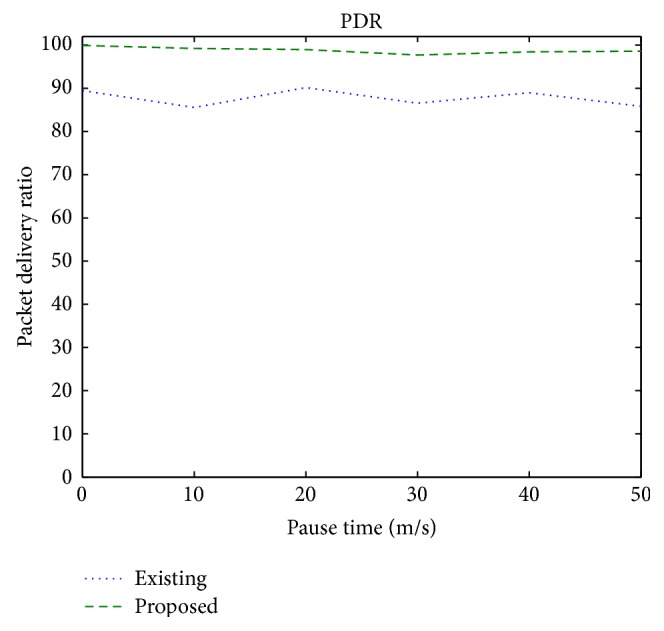
Packet delivery ratio versus pause time.

Figure 3 shows the packet delivery ratio under different pause times. It is observed that the proposed scheme achieves 11% increase in the PDR in a mobile environment. This is due to the fact that mobile nodes may cause packet losses, which in the proposed scheme is overcome due to the availability of the forwarding candidate nodes.

**Figure 4:**
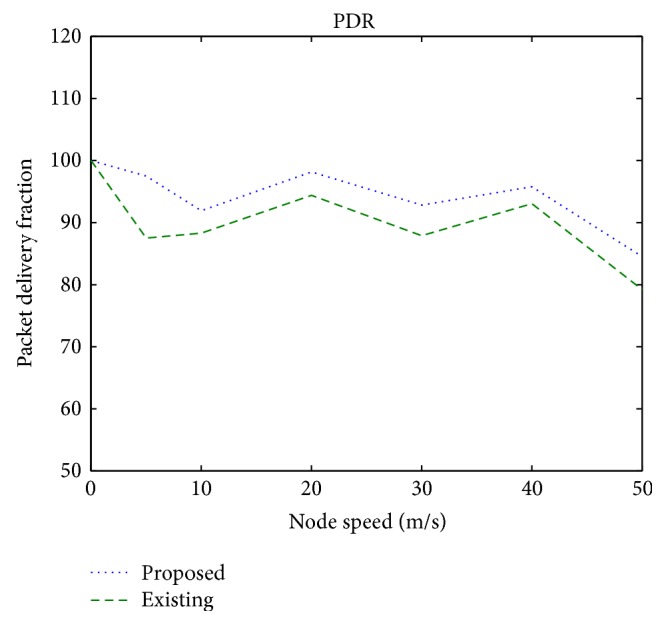
Packet delivery ratio versus node speed.

Figure 4 shows the packet delivery ratio under different node speeds considering the mobile nature of the wireless ad hoc network. It is observed that the proposed scheme achieves 6% increase in the PDR in a mobile environment.

### 3.2. Normalized Routing Overhead

It is defined as the ratio of the control packets transmitted to the total number of data packets delivered to the destination.

**Figure 5:**
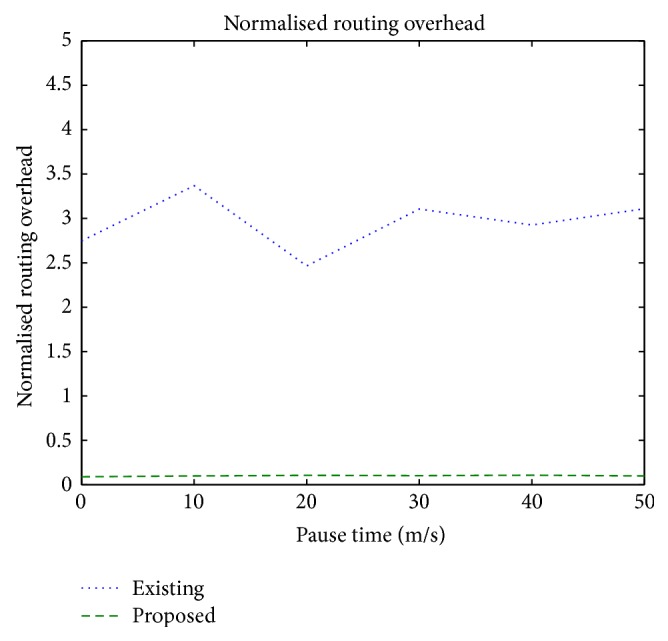
Normalized routing overhead versus pause time.

Figure 5 shows the routing overhead incurred due to route discovery and route rediscovery processes. Since the proposed scheme does not use control packets after link failure, the proposed scheme has, on an average, low overhead of 9%, whereas the traditional scheme incurs a high overhead of 29%.

### 3.3. Packet Drop Ratio

It is the total number of packets dropped out of the total number of packets generated by the sources.

**Figure 6:**
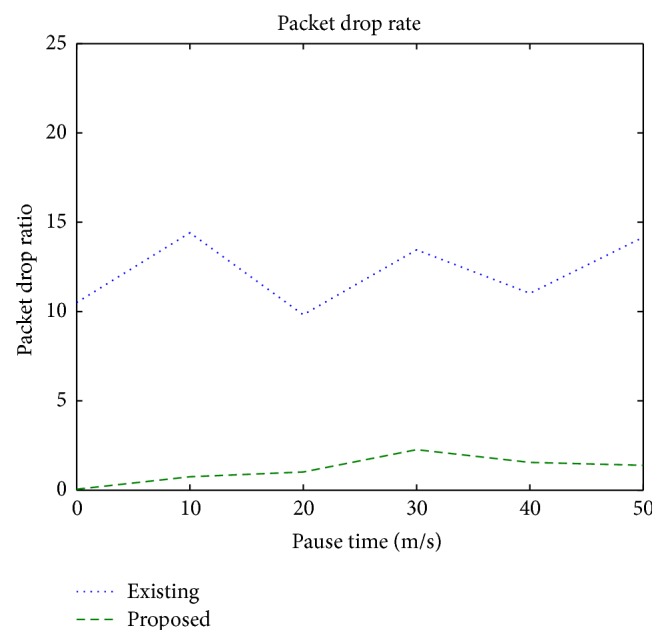
Packet drop ratio versus pause time.

Figure 6 shows the packet drop ratio when the nodes are made mobile with different pause times. The packet drop in the proposed protocol is 1% due to the selection of link quality aware optimal routes and the link monitoring, as against the 12% of existing protocol.

### 3.4. End-to-End Delay

It is the time interval between the generation time at the source and the arrival time of the packets at the destination and includes buffering of packets, route discoveries, and transmission, retransmission, and propagation delays.

**Figure 7:**
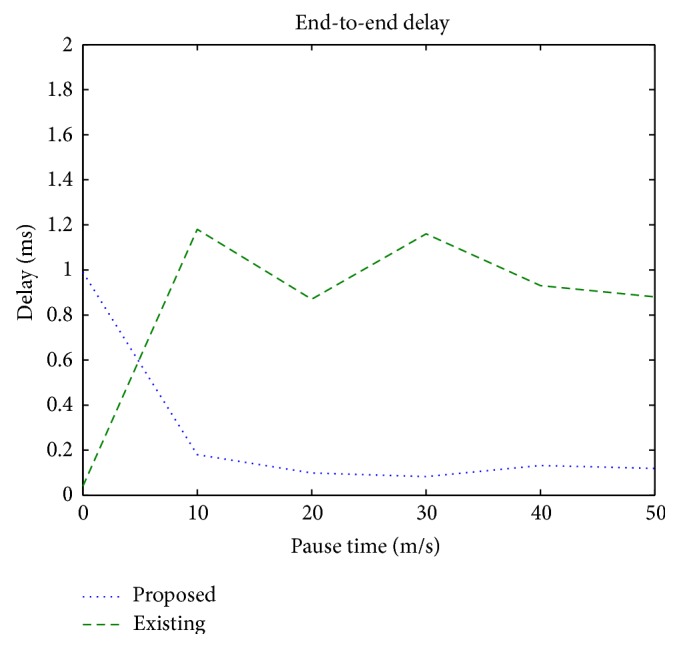
End-to-end delay versus pause time.

Figure 7 illustrates that the end-to-end delay incurred in a mobile network, using the proposed protocol, is appreciably less than that incurred in the existing protocol.

### 3.5. Total Energy Consumption

It is the total energy consumed in the network for the transmission of all the packets from source to destination.

**Figure 8:**
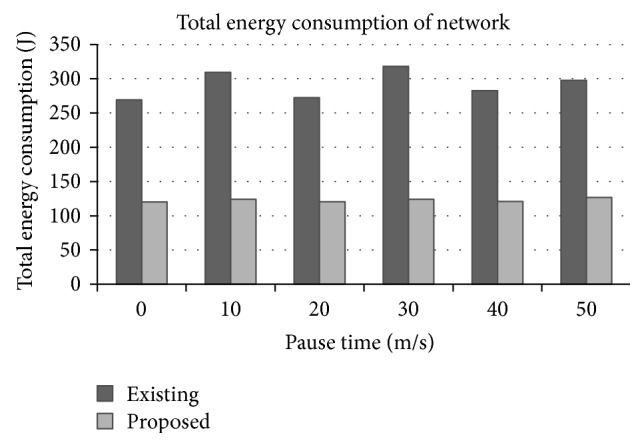
Total energy consumption in the network versus pause time.

Figure 8 shows the total energy consumed in the network for the packet transmission while varying the pause time of the mobile nodes. it is found that there is considerable energy saving of around 58% because of the selection of relay nodes based on link quality and variable transmit power levels. This in turn improves the lifetime of the network and therefore there is increased traffic carrying capacity.

### 3.6. Average Residual Energy

It is the amount of energy available with the nodes at the end of packet transmission. It is an important parameter for monitoring the lifetime of the battery powered mobile network.

**Figure 9:**
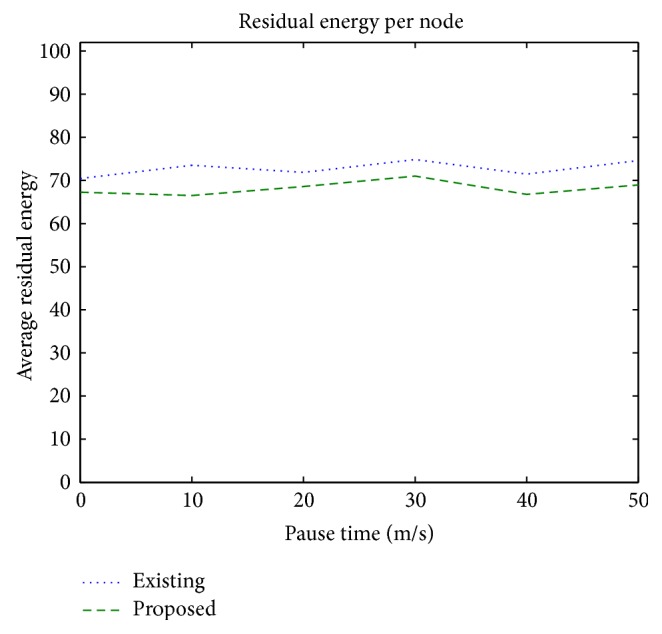
Average residual energy versus pause time.

Figure 9 shows that the average residual energy available with the mobile nodes at the end of the transmission is higher by 5% using the proposed protocol when compared to the existing protocol.

### 3.7. Network Lifetime

**Figure 10:**
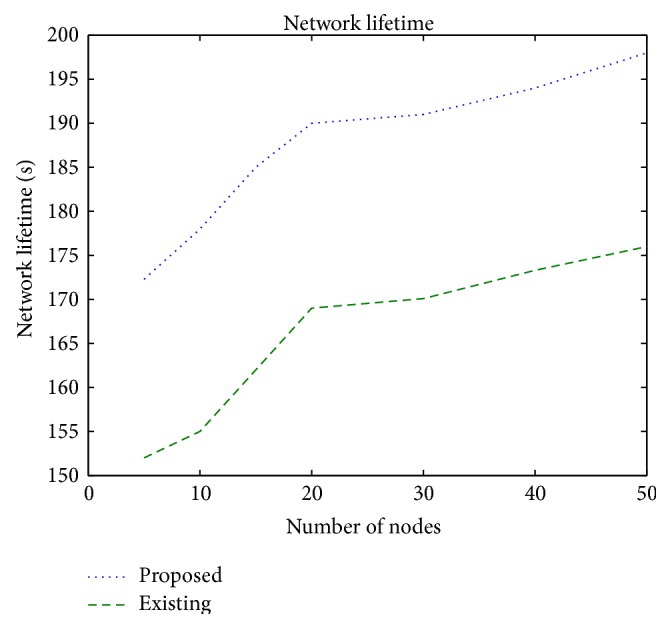
Network lifetime versus number of nodes.

The network lifetime is a measure of the network disconnectivity when a node is completely depleted of its energy. Since the proposed protocol considers the energy level of the node while making the relay node selection, the scheme achieves increased network lifetime when compared to the existing scheme.  Figure 10 shows that the network lifetime increases with increase in the network size in the proposed scheme due to knowledge of the residual energy and link state of the nodes. When the number of nodes is less from 5 to 15, the network lifetime increases but there is no appreciable change when the node numbers increase rapidly. The proposed protocol achieves 12% improvement when compared to the existing protocol.

### 3.8. Execution Time

**Figure 11:**
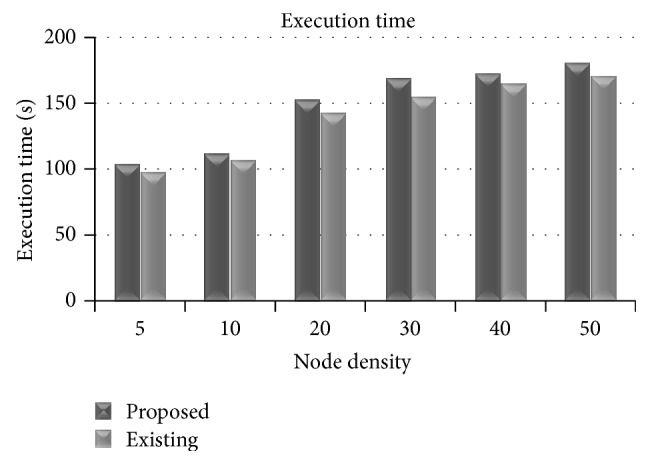
Execution time versus node density.

Figure 11 shows the difference in the execution of the two protocols.

The proposed protocol requires more resources for computation but the difference is very marginal. The highest change (about 5%) in execution time occurs when the node density is large. This may be contributed due to the selection of relay nodes according to the multiple metrics instead of the single metric computation. As node density increases, more nodes are available as relay nodes and the computation increases.

From all the comparative studies carried out, it is shown that the proposed approach achieves significant improvement in the performance of the wireless network in a mobile environment.

## 4. Conclusion

This paper proposes a new Energy Efficient Link Quality Aware Routing Protocol with Variable Power Control (ELRPP) for wireless ad hoc networks. It uses the concept of discovering routes based on the energy level and the instantaneous link quality. A variable transmit power level has been implemented and the evaluation using simulations shows that the proposed protocol improves the network performance in terms of various QoS parameters when considering the multiple metrics for routing when compared to the existing single metric based routing protocol.
